# Suicide in deaf populations: a literature review

**DOI:** 10.1186/1744-859X-6-26

**Published:** 2007-10-08

**Authors:** Oliver Turner, Kirsten Windfuhr, Navneet Kapur

**Affiliations:** 1 National Confidential Inquiry into Suicide and Homicide by People with Mental Illness, Centre for Suicide Prevention, University of Manchester, Manchester, UK

## Abstract

**Background:**

Studies have found that deaf individuals have higher rates of psychiatric disorder than those who are hearing, while at the same time encountering difficulties in accessing mental health services. These factors might increase the risk of suicide. However, the burden of suicidal behaviour in deaf people is currently unknown.

The aim of the present review was to provide a summary of literature on suicidal behaviour with specific reference to deaf individuals. The objectives of the review were to establish the incidence and prevalence of suicidal behaviour in deaf populations; describe risk factors for suicidal behaviour in deaf populations; describe approaches to intervention and suicide prevention that have been used in deaf populations.

**Methods:**

A number of electronic databases (e.g. Medline, PsycINFO, CINAHL, EMBASE, Dissertation Abstracts International, Web of Science, ComDisDome, ASSIA, Education Sage Full Text, Google Scholar, and the grey literature databases FADE and SIGLE) were explored using a combination of key words and medical subject headings as search terms. Reference lists of papers were also searched. The Science and Social Sciences Citation Index electronic databases were used to identify studies that had cited key papers. We also contacted experts and organisations with an interest in the field.

**Results:**

Very few studies focussed specifically on suicide in deaf populations. Those studies that were included (n = 13) generally involved small and unrepresentative samples. There were limited data on the rate of suicidal behaviour in deaf people. One study reported evidence of hearing impairment in 0.2% of all suicide deaths. Another found that individuals with tinnitus seen in specialist clinics had an elevated rate of suicide compared to the general population. The rates of attempted suicide in deaf school and college students during the previous year ranged from 1.7% to 18%, with lifetime rates as high as 30%. Little evidence was found to suggest that risk factors for suicide in deaf people differed systematically from those in the general population. However, studies did report higher levels of depression and higher levels of perceived risk among deaf individuals than hearing control groups. No firm evidence was found regarding the effectiveness of suicide prevention strategies in deaf people, but suggested strategies include developing specific screening tools, training clinical staff, promoting deaf awareness, increasing the availability of specialist mental health services for deaf people.

**Conclusion:**

There is a significant gap in our understanding of suicide in deaf populations. Clinicians should be aware of the possible association between suicide and deafness. Specialist mental health services should be readily accessible to deaf individuals and specific preventative strategies may be of benefit. However, further research using a variety of study designs is needed to increase our understanding of this issue.

## Introduction

Suicide and self-harm are major problems worldwide [1]. In the general population, mental illness is a major risk factor for suicide [2]. Deaf and deaf-blind individuals suffer higher rates of mental health problems than hearing individuals [3,4]. Recent reports from the UK Department of Health and non-governmental organisations [3,5] also reveal increased difficulties for deaf people in accessing mental health and social care services. These factors may put deaf individuals at greater risk of suicide than the general population.

Currently, the scale of the problem is unknown. The estimated number of people in the UK who are deaf or hearing impaired is 9 million (Royal National Institute for the Deaf statistics, as of 17 November 2006 [6]). If the rate of suicide in deaf people is no greater than in the hearing population (around 10 per 100000 per year) we might expect approximately 900 suicides per year by those who are deaf or hearing impaired. Based on the rates of self-harm in the UK general population (i.e. around 300–500 per 100000 per year [7] we might expect between 27000 and 45000 self-harm presentations to hospital each year by those who are deaf or hearing impaired. The size of the deaf and hard of hearing population in the United States has been estimated as around 20 million [8]. So in the United States, if the rate of suicide in deaf people is no greater than in the hearing population (around 11 per 100000 per year; US Government statistics, as of 16 April 2007 [9]) we might expect approximately 2000 suicides per year by those who are deaf or hearing impaired.

Risk factors for suicide in deaf and hearing-impaired people are also unknown. Some authors have suggested that risk factors for poor mental health are similar in the deaf and hearing populations [10,11]. Others have highlighted risk factors that are more specific to deaf populations, for example lack of role models, alienation from family and peers [12].

### Definitions

Terms used to describe both suicidal behaviour and deafness vary. Suicide describes an intentional act which has resulted in death [2]. For the purposes of this study we were also interested in non-fatal suicidal behaviours, for example attempted suicide (which implies a degree of suicidal intent and is a term particularly used in North America [13], and self-harm (an act of intentional self-poisoning or injury irrespective of the apparent purpose of the act [14]. We also included suicidal ideation [13].

A variety of terms are used to describe people with hearing loss [6]. For example, 'hard of hearing' may be used to describe people who have lost their hearing gradually, 'deafened' refers to people who were born hearing and became severely or profoundly deaf after learning to speak. Deafness may be further classified according to the degree of hearing impairment (mild, moderate, severe, profound). One important distinction is between those whose preferred method of communication is Sign Language (who may be pre-lingually deaf, and may refer to themselves as 'Deaf' (with a capital 'D') to emphasise their deaf identity) and those with hearing loss who use oral methods of communication. For the purposes of this review, we were interested in all forms of deafness.

### Objectives

An understanding of the epidemiology and risk factors for suicide in deaf people is essential to inform preventative initiatives. The current review had three main objectives:

• to establish the incidence and prevalence of suicidal behaviour in deaf populations;

• to describe risk factors for suicidal behaviour in deaf populations (demographic, clinical, service related etc.);

• to describe approaches to intervention and suicide prevention that have been used in deaf populations.

An additional objective of the review was to investigate subgroups of deaf people (for example those deaf from birth and those with later-onset deafness) with specific reference to each of the above objectives.

## Methods

We performed searches for literature in the key areas of suicide and deafness, primarily through electronic databases. These were: Medline (1966 to date); PsycINFO (1806 to date); CINAHL (1982 to date); EMBASE (1974 to date); Dissertation Abstracts International (1861 to date); Web of Science (1945 to date); ComDisDome; ASSIA (1987 to date); Education Sage Full Text (1968 to date) and the 'grey' literature databases FADE, and SIGLE. In addition, the internet search engine Google Scholar was explored.

The key search terms of 'deafness' and 'suicide' were entered into the databases, along with truncated versions and related terms, e.g. 'deaf$', 'hear$' and 'suicid$' to ensure a broad sweep for relevant papers. Terms relating to other hearing conditions, including Ménière's disease were also entered, as well the broader term of 'hearing impairment'. A variety of terms for suicidal behaviour were also used, for example 'attempted suicide', 'parasuicide', 'self-harm' and 'self-injurious behaviour'. A combination of medical subject headings and key word searches were performed. Finally, advice on additional terms was sought from our project collaborator, the Deputy Chief Executive of the deafness and mental health charity, Sign.

We also searched reference lists of retrieved papers to identify any other relevant studies, and explored the Science and Social Sciences Citation Index databases to identify studies that had cited key papers. Efforts were also made to contact experts in the field, particularly those involved in the deaf community. Two national conferences organised by the British Society for Mental Health and Deafness (BSMHD) were also attended during 2006 with the aim of locating additional unpublished work. Additional file 1 gives details of our search strategy.

We included studies where the participants were either deaf (complete hearing loss) or partially deaf (hearing impaired). We limited the study to English language papers and excluded case studies with less than five subjects and research correspondence that did not report empirical data. We excluded any literature published before 1966. The coverage of one of our principal databases (Medline) did not extend to the years prior to this. It was also felt that changes in the epidemiology of suicide, changes to mental health service provision and changes to the services for deaf people in the intervening period would have made the findings from older studies less relevant to current practice. However, we acknowledge that even in the period since 1966, some of the changes to practice have been very significant. In any case, research from countries with private health care systems (for example, the US) may have limited relevance for practice in countries with managed health care systems (for example the UK). We also considered studies relating to tinnitus and other sensory impairments if they were relevant to the study aims. Data were extracted independently by two raters using structured proformas. We chose not to formally rate the methodological quality of available literature with checklists or quality scales. Instead, the strengths and weaknesses of each individual study were commented on in detail.

## Results

A total of 1005 papers were identified by the search strategy. Of them, 131 were excluded on the basis of their titles alone and a further 787 were excluded on the basis of their abstracts. Full text versions were retrieved for 87 papers and 13 were considered directly relevant to the objectives of the review (Table 1). The studies were published between 1986 and 2006. Eight studies recruited samples from the USA, two from the United Kingdom, one from Australia. The remaining two studies were based on international samples. The reliability of data extraction was excellent, with no disagreements between raters with respect to key data.

**Table 1:** Suicide in deaf populations: studies included in this review

**Author**	**Design**	**Aims**	**Participants/setting**	**Main outcome measures**	**Key findings**
Black and Glickman, 2006	Prevalence study	To examine demographic and clinical characteristics of deaf and hearing psychiatric in- patients	A total of 64 deaf adult patients of specialist deaf unit at Westborough State Hospital, USA. All discharged between 1999 – 2004 (55% male; 45% female). No mention of age. Controls: 64 hearing patients discharged between 1999 – 2004. A total of 180 hearing patients seen on one day in March 2006.	The Clinical Evaluation of Risk and Functioning Scale, revised. The Allen Cognitive Levels Scale. Language Rating Scale.	Deaf psychiatric inpatients rated at significantly higher risk of self-harm than hearing psychiatric patients by clinicians. A total of 23.4% of deaf patients diagnosed with major depressive disorder.
Boyechko, 1992	Prevalence study	To explore attitudes, experience and associated risk factors for suicide among hearing impaired college students. To explore the relationship between suicidal behaviour and depression, hopelessness and social support.	60 deaf college students of Gallaudet University, Washington DC, USA, recruited via personal appeal and by post. Nine excluded for recording 'outlying results.'	Suicide Opinion Questionnaire, Suicide Information Questionnaire, Provision of Social Relations, Beck Depression Inventory Hopelessness Scale.	Over lifetime: 30% attempted suicide, 30% seriously considered suicide. During past year: 18% attempted suicide, 18% seriously considered suicide. No completed suicides.
Critchfield et al, 1987	Survey	To determine the types and levels of suicide intervention techniques in place at various educational settings for the deaf and hearing impaired. To investigate frequency of suicidal behaviour over 1 year period in these settings.	A total of 92 (from 153 approached) US schools for the deaf; 45% were deaf-only programmes, 31% combined deaf/hearing, 24% deaf post-secondary. A total of 45% had residential students, 55% had day students. All students of junior high school age or above.	Questionnaire (not provided) concerning frequency of suicidal 'attempts/gestures'; verbalization and hospitalisation of students.	A total of 503 (6.3%) incidents of suicidal behaviour during past year among 8 020 students; 134 suicidal attempts/gestures (1.7%), 69 hospitalisations for suicidal or depressive episode. No completed suicides.
De Leo et al, 1999	Prevalence study	To investigate the physiological and pathological reactions to sight loss. To understand pathological reactions to fear of going blind. To investigate a population of suicides involving the fear of blindness.	A total of 3 654 autopsy case reports for suicide over the period 1990 – 1997 in Queensland, Australia.	Coroner's post-mortem reports, detailing: age; gender; psychiatric history; major life changes resulting from impairment; social and family support and other events. Police reports and questionnaires. Cases included if there was mention of visual/hearing impairment in coroners' records.	A total of 19 cases (0.52% of sample) found to have sensory impairment. Twelve cases (0.3% of sample) found to be visually impaired. Seven cases (0.19% of sample) found to be hearing impaired. In two cases, hearing loss described as 'major contributing factor' to suicide.
Dudzinski, 1998	Survey	To explore the presence, perception and impact of suicidal ideation in deaf students (focus on young adults). To assess response patterns to suicidal behaviour in deaf schools.	A total of 42 (from 83 approached) US residential and day educational programmes for the deaf. No information on attending students (e.g. age, gender).	Eight-item suicide ideation questionnaire, completed by principals, associate principals, senior counsellors and supervisors.	Estimated prevalence from results: 8% of all students were referred to counselling for suicidal ideation during academic careers, representing approximately 17% of all students referred to counselling.
Jacobsen and McCaslin, 2001	Literature review	To establish evidence of a direct relationship between tinnitus and suicide.	Clinical populations	Medline and HealthStar electronic databases, using search terms 'tinnitus' and 'suicide'.	Four articles found directly addressing tinnitus and suicidal behaviour. No evidence of causal relationship.
Leigh et al, 1988	Cross-sectional study	To modify the Beck Depression Inventory for use with the deaf.	A total of 214 college students: 112 hearing, 102 deaf (students at National Technical Institute for the Deaf, USA.) Hearing loss > 80 db. No mention of participant selection methods.	Beck Depression Inventory. Beck Depression Inventory, revised for use with the deaf.	Mean BDI: 10 (deaf); 7.8 (hearing). No difference among hearing group in scores of original and revised test versions. Lower internal consistency in scores of revised version among deaf than hearing students.
Leigh et al, 1989	Cross-sectional study	To investigate whether deaf and hearing populations differ in experiences of depressive symptoms.	A total of 214 college students: 112 hearing, 102 deaf (students at National Technical Institute for the Deaf, USA). Hearing loss > 80 db.	Beck Depression Inventory. Sociotropy-Autonomy Scale. Parental Bonding Instrument. All revised for use with the deaf.	A total of 43% of deaf students compared to 27% of hearing students scored within range of mild depression; 8% of deaf students compared to 4% of hearing students scored within range of moderate depression.
Lewis et al, 1992	Case study	To investigate tinnitus as a possible risk factor for suicide. To consider additional risk factors for suicide.	Six case studies of suicidal behaviour in tinnitus sufferers known to one clinic in Cardiff, Wales (Mar 1990 – April 1991).	Case reports of suicide, detailing social and demographic information, psychiatric history and type and severity of tinnitus.	Suicide rate: 118 per 100 000 for clinic attenders with tinnitus .Suicide risk factors: male gender; low SE class; social isolation; depression and other psychological problems.
Lewis et al, 1994	Survey	To inform practitioners of the risk factors for suicide among tinnitus sufferers.	A total of 50 audiology clinics contacted worldwide, from which 17 practitioners responded. A total of 23 cases of suicide in tinnitus sufferers, with five additional cases known to researchers.	A 20-item tinnitus and suicide questionnaire, requesting social and demographic information, psychiatric history and type and severity of tinnitus.	Suicide risk factors: male gender; low SES class; social isolation; bereavement; depression. A total of 90% of suicides in those aged > 40 years; 50% died within 2 years of onset of tinnitus.
Lewis and Stephens, 1995	Prevalence study	To determine the rate of attempted suicide among tinnitus sufferers.	A total of 184 patients admitted to poisons unit of one hospital in Glamorgan, South Wales.	A five-item tinnitus questionnaire, eliciting information on type, severity and duration of condition.	Three cases of tinnitus in 184 patients, representing 1.6% of entire sample. (General population prevalence of tinnitus around 7%.)
Marcus, 1991	Prevalence study	To provide a videotaped version of the Beck Depression Inventory in American sign language. To investigate the frequency of depression among deaf college students.	Experiment 1: 28 deaf college students. Experiment 2: 129 deaf college students. All paid volunteers from Gallaudet University, Washington DC.	Beck Depression Inventory. Brauer – Gallaudet Beck Depression Inventory (BGBDI; videotaped in American Sign Language for use with the deaf). MMPI-depression scale (videotaped in American Sign Language for use with the deaf).	Average score on BGBDI of 14.1; 61% had some depressive symptoms, 35% scored within range indicating mild depression. A total of 19% scored within range indicating moderate to severe depression; 7% scored within range indicating severe depression
Watt and Davis, 1991	Cross-sectional study	To explore the relationship between boredom-proneness and depression in deaf residential school students.	A total of 110 college students: 50 deaf (residential school), 60 hearing (junior high school) in south-eastern Unites States.	Boredom Proneness Scale. Beck Depression Inventory. Two versions of each: original and revised (for use with deaf).	A total of 40% of deaf vs 17% of hearing students recorded mild depression; 6% of deaf vs 3% of hearing students recorded moderate depression. Deaf students significantly more boredom-prone than hearing students.

### Incidence/prevalence of suicidal behaviour

Six papers offered varying amounts of information relating to the incidence and prevalence of suicidal behaviour in deaf people.

Critchfield, Morrison and Quinn surveyed 153 US schools and educational programmes for deaf students [12]. The aim was to gather information on the frequency of suicidal behaviour in hearing-impaired adolescents during the previous year. It should be noted however that the terms 'deaf' and 'hearing impaired' were used interchangeably throughout the paper and no definitions related to the hearing status of students were offered. Results were obtained from questionnaires administered to educational environments either exclusively for deaf students or which had at least 100 deaf students attending. In total, 92 institutions (covering a total of 8020 students) responded. The researchers found that, overall, 1.7% of students had made suicidal attempts or 'gestures' (referring to acts of suicidal behaviour with uncertain intent) during the previous year. No completed suicides were reported. It is difficult to comment on how generalisable these results are to the wider deaf population. The results were based on students of junior high school age or above in selected institutions. In addition, 61% of questionnaires were returned and no information was reported on those institutions which did not participate.

Boyechko reported prevalence rates of suicidal behaviour in hearing-impaired (hearing loss of 55 decibels or greater) US college students at a single specialist higher education institution [11]. A total of 51 deaf individuals completed study questionnaires. The author found high rates of suicidal behaviour and ideation among participants. During their lifetime, 40% reported having felt that life was not worth living and 44% had experienced suicidal thoughts. Overall, 30% reported having attempted suicide during their lifetime and 18% had attempted suicide during the previous year. There were no completed suicides. The study had several methodological limitations. First, the sample size was relatively small and only comprised students from a single specialist University for deaf students. Second, the sample comprised volunteers who may have had a specific interest in suicide and may therefore not have been representative of the general population. Third, the study sample was recruited in two different ways, the first through an appeal for participants during university lecture sessions and with posters displayed around the university campus, the second by inviting students to participate by post.

Dudzinski surveyed all US residential and day education programmes for deaf students listed in the American Annuls of the Deaf Directory (n = 83) to investigate suicidal ideation in deaf people [10]. The study was also designed to analyse administrative response patterns to expressions of suicidal ideation. No exact incidence rates were presented but from the study results it is possible to estimate that approximately 8% of all students at the institutions were referred to counselling for suicidal ideation (approximately 17% of all students referred to counselling services). Methodological weaknesses included possible response bias; only 51% of institutions completed the survey. The study also used a brief self-report data collection tool that had not been validated.

De Leo and colleagues investigated reports of visual impairment and deafness in all 3654 autopsy case reports of suicide in Queensland, Australia collected for the period 1990 – 1997 [15]. Twelve individuals were described as 'sight impaired'. Seven were described as 'hearing impaired'. This later group included three cases of tinnitus, two cases of Ménière's disease, and two cases of 'deafness'. (These terms were not expanded upon, perhaps because all data was collected from retrospective coroner's reports). The 19 cases reported by De Leo et al represented 0.5% of the sample, with those with hearing impairments representing 0.19%. Hearing impairment was considered a direct contributing factor to suicide in two of the suicide cases (one suffered tinnitus and one suffered Ménière's disease). The authors examined a large number of autopsy reports. However, the small number of cases and the lack of denominator data make it difficult to draw definitive conclusions about the incidence of suicide in deaf populations. De Leo et al's figures may also have underestimated the incidence of suicide in deaf people as sensory impairments were only identified if mentioned in the coroners' reports.

Two papers examined the relationship between tinnitus and suicide. Lewis et al found that four individuals out of a clinic population of 674 in Cardiff, Wales had died by suicide over a 5 year period; a rate of 118 per 100000 per year [16]. This was over 10-times the general population rate of suicide in South Glamorgan (the County including Cardiff) during this time. The study presented the case histories of the tinnitus sufferers who had completed suicide (and two additional case histories: one patient who was not a clinic patient but had tinnitus and died by suicide, and one clinic patient who had been killed by his son but who had attempted suicide the previous week). By the authors' own admission, firm conclusions regarding true rates of suicide in tinnitus sufferers are difficult to determine based on a limited number of cases. Also, as all individuals were identified from a specialist clinic population, these cases may have had the most severe tinnitus and may not have been representative of tinnitus sufferers more generally.

Lewis and Stephens surveyed 184 patients of a poisons unit of one hospital in South Glamorgan, Wales during the 3-month period August to October 1993 [17] and achieved a 100% response rate. The authors reported that tinnitus sufferers were under-represented in the self-poisoning population (3 patients; 1.6%) compared to the general population (7%), perhaps because South Glamorgan has a well-developed service for providing help to those with tinnitus, or those with tinnitus harm themselves in ways other than self-poisoning. The 3-month time period allowed for a very limited sample of patients and the study did not consider tinnitus sufferers who may have poisoned themselves and simply not presented for treatment, or presented to other hospitals or primary care.

### Risk and associated factors for suicide in deaf people

Eight studies attempted to describe possible associations with suicide.

Critchfield et al found a higher rate of suicidal attempts and 'gestures' among deaf students at deaf-only educational programmes (2.2%) than among deaf students at combined deaf and hearing programmes (0.9%) [12]. The same applied for verbalisation of suicide (4.6% in deaf-only programmes; 2.7% in combined deaf and hearing programmes) and hospitalisation for a suicidal or depressive episode (1% in deaf-only programmes; 0.6% in combined deaf and hearing programmes).

Boyechko found that suicidal behaviour was associated with decreasing levels of hopelessness, and decreasing levels of social support [11]. Dudzinski reported that the two most common reasons for considering suicide among both deaf males and females were family and relationship problems [10].

Lewis, Stephens and McKenna obtained details of 28 cases of suicide in tinnitus sufferers by surveying audiology clinics in the UK and other developed countries [18]. From these cases Lewis et al identified a number of common risk factors for suicide, including male gender, social isolation, a history of psychiatric illness, problems with alcohol use and a prior history of attempted suicide. The sample was small but the study provided interesting results.

De Leo et al found that of the seven cases of suicide by hearing-impaired individuals, hearing-related problems had been a major contributing factor in two cases (29%). A total of 63% had a history of mental illness and 43% had experienced a recent traumatic event prior to suicide [13].

In our search of the deafness and depression literature we found no studies which included suicidal behaviour as a main outcome measure. However, Leigh et al piloted and modified the Beck Depression Inventory (BDI) for use with deaf people (with a hearing loss of 80 decibels or greater) [19]. They reported high internal consistency for the revised BDI in hearing individuals, similar to that reported for the original BDI (Cronbach's alpha 0.9). The mean scores on the two versions of the scale were also very similar. However, internal consistency was slightly lower for deaf individuals (Cronbach's alpha 0.7). They found that 102 deaf students recorded a mean score of 10 on the revised BDI. This compared to a mean score of 7.7 for 112 hearing students on the original version. Leigh et al reported additional findings from the same study in a later paper [20]. On the revised BDI, 43% of deaf students scored within the range indicative of mild depression, compared to 27% of hearing students, and 8% of deaf students scored within the range indicative of moderate depression, compared to 4% of hearing students. Although the findings of this study are suggestive of higher BDI scores (indicating more severe depressive symptoms) among deaf compared to hearing students, the authors emphasised that they did not feel that the revised version of the BDI was ready for clinical use. It was still at a research and development stage and further work on its psychometric properties in different groups of deaf individuals was needed.

Similar results were obtained from a study of 50 profoundly deaf (hearing loss 90 decibels or greater) residential school adolescents and 60 hearing adolescents from a junior high school carried out in the south-eastern United States [21]. The mean score on the revised BDI was 10.5 for deaf students compared to 6.6 for hearing students. Overall, 40% of deaf students scored within the range indicative of mild depression, compared to 17% of hearing students, and 10% of deaf students scored within the range indicative of moderate or severe depression, compared to 3% of hearing students. However, because of the different routes of recruitment for deaf and hearing students it is difficult to be certain that the between-group differences are robust.

Marcus tested 129 deaf college students using a videotaped version of the Beck Depression Inventory (the Brauer-Gallaudet Beck Depression Inventory; BGBDI) [22]. The paper did not give a definition of the term 'deaf' nor discuss the severity of hearing loss in those who participated. Overall, 61% of students recorded scores indicative of significant depressive symptoms. A total of 35% recorded scores indicating mild to moderate depression, a further 19% recorded scores indicating moderate to severe depression, and 7% scored within the severe depressive symptoms range. The hearing condition of participants' parents was also identified as a risk factor for depression. Deaf participants with deaf parents recorded a mean score of 10.4. Deaf participants with hearing parents recorded a mean score of 15.3. This difference was statistically significant (p < 0.05).

Black and Glickman compared the clinical characteristics of deaf patients discharged from one specialist deaf psychiatric inpatient unit to those of hearing patients from the same hospital [23]. The 'deaf' group in this study was described as containing those who were both 'deaf and 'severely hard of hearing', although no further explanation related to these terms was offered. Black and Glickman found a wider range of diagnoses among the deaf patients than had been reported in previous studies. Post-traumatic stress disorder was the most common diagnosis (29.7% of deaf patients and 21% of hearing patients). Affective disorder was more common in deaf than hearing patients (26% of deaf patients compared to 8.3% of hearing patients) but psychotic and substance misuse disorders were less common. Importantly for this review, deaf patients were considered by staff as at significantly higher risk of self-harm than hearing patients. The mean scores (standard deviations) on the self-harm section of the Clinical Evaluation of Risk Functioning Scale were 3.3 (1.5) for deaf patients, compared to 2.1 (1.3) for hearing patients. The study sample was small (n = 64 deaf in-patients) and taken from a single unit of one hospital. The findings are therefore difficult to generalise to the wider deaf population. In addition, in-patient units for the deaf may function very differently from in-patient units for hearing patients, making direct comparisons between them difficult. The study does however give an interesting insight into the prevalence of a broad range of psychiatric diagnoses in a deaf population in a clinical setting.

### Approaches to suicide prevention

We found no studies that formally evaluated existing suicide prevention strategies for use with deaf people, but a number of studies reported material relevant to this aim.

Dudzinski reported that although most participating schools for deaf students considered suicidal behaviour a problem, almost one-third (31%) had no established guidelines for responding to such behaviour [10]. Further, the five most common elements of procedures for dealing with suicidal ideation listed by Dudzinski appear generic and applicable to deaf and hearing students: (1) call parents, (2) keep student under observation, (3) complete written documentation, (4) call counsellor/psychologist, (5) follow-up. In schools with policies for dealing with suicidal ideation, the most common response type was administrative, e.g. contacting a supervisor or writing a report of the event [10]. In some schools the policies were exclusively administrative in nature. The least common intervention was psychosocial.

Critchfield et al found that from 92 schools and residential programmes for deaf students or deaf and hearing students, only 21% had an established policy for dealing with suicidal behaviour [12]. The authors also presented a suicide intervention model used at the California School for the Deaf in Fremont (CSDF), California. The model suggests a long-term treatment plan for students displaying suicidal behaviour and a checklist for counsellors to aid their evaluations of students referred for suicidal ideation. The CSDF has also created a comprehensive 'decision tree' detailing which types of involvement by staff are most appropriate, depending on the levels of suicidal ideation expressed by the student. Critchfield et al argue that the long-term treatment of suicidal behaviour in deaf adolescents should vary little from that for their hearing peers [12]. This is reflected in the suicide intervention model at the CSDF which reflects a generic approach to suicide prevention in students.

Lewis et al [16] suggested the need for an enquiry and possible psychiatric referral following risk assessment of tinnitus sufferers, and emphasised the role of clinicians in identifying tinnitus sufferers at risk from suicide [18]. Leigh et al modified the Beck Depression Inventory for use with deaf people [19]. This could be a useful tool for preventing suicidal behaviour by helping to identify an established risk factor for suicide.

### Subgroups of deaf people

We found little data regarding suicidal behaviour within subgroups of deaf populations. We were primarily interested in investigating whether there were any differences between those with pre-lingual deafness and those with later onset deafness. There was little consensus on definitions and the terms 'deaf' and 'hard of hearing' were often used interchangeably, highlighting the extent to which those with hearing impairments were considered a uniform group.

## Discussion

### Main findings in relation to aims

#### Incidence/prevalence of suicidal behaviour

De Leo et al reported that 0.2% of those completing suicide in one Australian state had a hearing impairment [15]. A small study of tinnitus sufferers presenting to one clinic suggested their suicide risk was 118 per 100000 per year (over 10 times the general population rate) [16]. Lifetime rates of attempted suicide among deaf and hearing-impaired school and college students were as high as 30% [12]. Rates of attempted suicide during the previous year ranged from approximately 1.7% [12] to 18% [11]. Methodological weaknesses mean that these estimates should be interpreted cautiously and it is unclear whether these rates are substantially higher than general population rates. Previous studies examining suicidal behaviour among unselected hearing student samples have reported rates of 1.7% during the previous year [24]. Studies of self-harm in hearing adolescents have reported annual rates of approximately 5% [25] to 7% [26,27]. Further studies using more robust methods would enable a better estimate of the risk of suicidal behaviours in deaf populations.

#### Risk and associated factors for suicide in deaf people

The literature suggests that a number of factors are associated with suicide in deaf and hearing-impaired people. However, it is difficult to comment on whether these are true risk factors as many studies were either descriptive or lacked adequate control groups. In general the factors associated with suicidal behaviour in deaf populations (for example, family and relationship problems, social isolation, psychiatric illness) were similar to the risk factors that have been reported in hearing populations [28]. We found little evidence to suggest that risk factors for suicide in deaf people differed systematically from those in the general population. However, studies did report higher levels of depression [18] and higher levels of perceived risk among deaf individuals than hearing control groups [23].

#### Approaches to suicide prevention

Surveys of educational establishments for deaf individuals appear to indicate only a minority have specific policies for dealing with the issue of suicidal behaviour. Suggested suicide prevention strategies for use in deaf people have largely relied on generic principles.

### Methodological limitations of the review

This review appears to be the first to report the available literature on suicide in deaf people but changes to our methodology may have increased the number of studies included. If translating resources had been available it may have been possible to include foreign language material (in the event only one foreign language study was found and this was of doubtful relevance). It may also be possible that more papers exist but are currently unpublished; some of the studies in this review were PhD dissertations rather than published papers. However, the study was advertised widely within the UK and this did not result in any additional material. Furthermore, we made contact with key researchers in the field and searched dissertation and 'grey' literature databases. A formal meta-analysis of studies may have strengthened conclusions of the review; however the limited number of relevant studies and their heterogeneity precluded this. We chose not to formally rate the quality of individual papers and discussed the strengths and weaknesses of individual studies instead. Alternative means of rating the data quality were not carried out. Finally, not all possible terms for depression related to deafness were explored. This is because it was not a central aim of the review. Rather, it was used to identify other papers relevant to the topic of suicidal behaviour in deaf people.

### Implications for future research

This review has highlighted a lack of research and the possibility that some aspects of suicidal behaviour may be more common in hearing-impaired individuals. Methodological limitations to previous studies include limited-response rates, small and unrepresentative samples, and unstandardised definitions. As discussed above, people with hearing loss are not a homogenous group. Studies have failed to distinguish between different types of deafness, different degrees of deafness, and those who use sign language compared to those who use oral methods of communication. Suicide is a rare event but future studies could utilise existing resources and databases, such as national suicide databases [29], and regional self-harm and attempted suicide databases [30]. Such databases may allow an estimate of the risk of suicidal behaviour among those who are deaf, especially if linked with general population databases [31,32]. Use of these databases would depend on whether baseline information on hearing status was available. We might also explore the use of databases in other countries in order to investigate the relationship between deafness and suicide. Such databases have been successfully used in Scandinavia to investigate the relationship between a number of risk factors and suicide (see, for example, [33]). Large-scale surveys could be used to recruit more representative samples of participants than have been recruited in studies to date by enlisting the help of organisations (such as the RNID in the UK) which work with a broad cross-section of deaf populations. Qualitative methodologies might help in the investigation of the processes underlying suicidal behaviour in deaf people and suggest possible avenues for prevention.

### Implications for practice

It is unclear whether rates of suicidal behaviour among deaf and hearing-impaired people are higher than in the general population. Deaf people may be more socially isolated, may have more physical health problems, and may be more likely to suffer depressive symptoms than those who are hearing [3]. These factors all increase the risk of suicide in the general population [28], and so it may be that deaf people are at greater risk of suicide than their hearing counterparts. Clinicians should be aware of this possibility. Even if the risk is not significantly increased in deaf individuals, services should still be accessible to this group and specific preventative strategies are likely to be of benefit. There is little firm evidence for the effectiveness of suicide prevention strategies in deaf people, but a number of practical options have been suggested by recent reports and policy documents.

A key concern is why more clinicians do not come into contact with those from the deaf community. A report from the UK Department of Health 'A Sign of the Times' [3] makes clear the need for increased accessibility for deaf people to mental services in England. One of the main messages of the document is that a 'one size fits all' approach to mental health services does not offer deaf and hearing-impaired patients an equal standard of care, and may discourage those from the deaf community from seeking help. The report outlines a number of measures aimed at improving access to mental health services for deaf people, including the training of clinical staff in deaf awareness and promoting the recognition of mental health problems in those who are deaf. Developing specific diagnostic and screening instruments for depression in deaf people may help to do this.

'Mental Health and Deafness – Towards Equity and Access' [34] suggests that 'Better community support, access to specialist care, and improved provision in prisons should all contribute to a reduction in the risk of suicide.' Another report, 'Making Positive Connections' [5], also makes a number of recommendations for improving services with a view to suicide prevention. These include the initiation of a full research project to further our understanding of the risk of suicide in deaf people, the need for specialist mental health services to be made available to both deaf children and deaf adults, and funding to allow deaf people access to frontline community services and for their availability to be made known to those who are deaf.

## Competing interests

The author(s) declare that they have no competing interests.

## Authors' contributions

NK and KW designed the study and secured funding in partnership with Sign. OT contributed to aspects of study design. The main literature review was carried out by OT with supervision from NK and KW. Data extraction was carried out by OT and KW and checked by NK. OT and NK took a lead in interpreting the findings and jointly wrote the paper. KW contributed to the interpretation of data and commented on drafts.

## Supplementary Material

Additional file 1 Project search strategy. Click here for file
