# Supplementary material for: Suicide in deaf populations: a literature review
Source: Ann Gen Psychiatry. 2007 Oct 8;6:26. doi: 10.1186/1744-859X-6-26 (PMC2093933; doi:10.1186/1744-859X-6-26)
Supplement: Additional file 1 — Project search strategy. [file 1744-859X-6-26-S1.doc]

## Electronic searching

Database: Ovid Medline (1966 to present)

| **Search term** | **Hits** |
| --- | --- |
| 1. Deafness.mp. or exp Deafness/ | 22 863 |
| 2. Hearing loss.mp. or exp Hearing loss/ | 28 477 |
| 3. Deaf$.mp. or exp Hearing impaired persons/ | 43 734 |
| 4. exp Meniere's Disease/ or Menieres Disease.mp. | 5 239 |
| 5. Hearing impair$.mp. | 7 640 |
| 6. Terms 1 or 2 or 3 or 4 or 5 | 57 353 |
| 7. Suicide.mp. or exp Suicide/ or exp Suicide, attempted/ | 41 776 |
| 8. Suicid$.mp. | 44 265 |
| 9. Attempted suicide.mp. | 2 440 |
| 10. Parasuicide.mp. or exp Self-injurious behavior/ | 37 079 |
| 11. Self harm.mp. | 896 |
| 12. Terms 7 or 8 or 9 or 10 or 11 | 48 198 |
| 13. Terms 6 and 12 | 95 |

Additional searches were conducted for:

- Blindness/other sensory impairments and suicide (using a variety of subject headings and key words, such as blindness; vision disorders; sight loss; sensory loss).
- Tinnitus and suicide (using tinnitus as a subject heading and key word).
- Deafness and depression (using a variety of subject headings and key words, such as depression and depressive disorder).

Other databases:

Dissertation Abstracts International

Web of Science

EMBASE

PsycINFO

CINAHL

ComdisDome

ASSIA

Education SAGE full text

FADE

Other electronic sources:

Google Scholar

## Reference searching

Databases:

Social Sciences Citation Index

Science Citation Index

Other:

Reference lists

## Personal contact

Selected authors and supervisors of published and unpublished studies were contacted, along with relevant professionals in the field, notably Dr Jim Cromwell, a chartered clinical psychologist at National Deaf Services, UK and Dr Peter Hindley, Chair of the British Society for Mental Health and Deafness.

The aim was to identify previously uncovered work. Two conferences on mental health and deafness were also attended.
